# Registered Nurses’ Perspectives on Documenting Social Determinants of Health in Electronic Health Records: A Qualitative Descriptive Study in Saudi Arabia

**DOI:** 10.3390/healthcare14213676

**Published:** 2026-10-30

**Authors:** Bader M. Almutairy, Waleed M. Alshehri, Abdulaziz M. Alodhailah, Mohammed Almutairi, Thurayya Eid

**Affiliations:** 1 Department of Community and Psychiatric Mental Health Nursing, College of Nursing, King Saud University, Riyadh 11451, Saudi Arabia; 2 Department of Medical-Surgical Nursing, College of Nursing, King Saud University, Riyadh 11451, Saudi Arabia

**Keywords:** social determinants of health, electronic health records, nursing documentation, nurses, qualitative research, health informatics, Saudi Arabia

## Abstract

Background/Objectives: Social determinants of health (SDOH) shape health outcomes, but their documentation in electronic health records (EHRs) remains inconsistent. Nurses capture social information during routine assessment, yet their perspectives on SDOH documentation are little studied outside North American and European settings. This study explored registered nurses’ perspectives on, and perceived barriers to, SDOH documentation in EHRs in Saudi Arabia. Methods: A qualitative descriptive study was conducted with 19 registered nurses purposively recruited with maximum variation from five governmental and private facilities in Saudi Arabia. Semi-structured interviews (35 to 75 min; mean 52 min) were conducted in Arabic or English between August and November 2025 and analyzed using reflexive thematic analysis in NVivo 14. Reporting follows the COREQ 32-item checklist. Results: Four themes were constructed: (1) system design and interoperability, with social history fields that were hard to locate, rigid response options, and limited data transfer across settings; (2) time as a competing resource, with social documentation deprioritized relative to medical documentation; (3) knowledge gaps and training needs, including uncertainty about what constitutes SDOH and about documentation protocols; and (4) from documentation to action, marked by a gap between recording social needs and acting on them. Conclusions: Participants valued SDOH documentation but described technological, temporal, educational, and organizational constraints. These barriers parallel those reported internationally, but in this setting, the pathway from documentation to action extends beyond the health system. Findings indicate a need for EHR design that places social data within clinical workflow, explicit protocols, targeted training, and referral pathways linking identified needs to services.

## 1. Introduction

Social determinants of health (SDOH) are the conditions in which people are born, grow, live, work, and age, and they shape health inequities through structural and intermediary pathways [1]. Empirical modeling of population health rankings attributes approximately 40% of modifiable determinants of health outcomes to social and economic factors, compared with 20% attributable to clinical care [2]. Housing instability, food insecurity, and social isolation are associated with barriers to obtaining care, poorer glycemic control, and increased mortality risk, respectively [3,4,5], yet systematic documentation of social circumstances in clinical settings remains inconsistent [6,7].

Electronic health records (EHRs) provide a mechanism for capturing SDOH data alongside clinical information [8], but analyses report substantial variability in what is captured and how it is structured [9,10]. Activating a structured screening tool does not by itself produce systematic documentation: across more than 100 community health centers, only 2% of patients with a visit had a documented SDOH screening [7], and where dedicated tools were implemented with support, only 19% of patients with a documented social need had a corresponding referral recorded in the EHR [11]. Contributing factors include EHRs designed around diagnoses and treatments rather than social circumstances [8], time constraints within clinical encounters [12], and limited training or uncertainty about clinicians’ role in addressing identified needs [13]. Responses have included ICD-10-CM Z codes, which remain underused [14], consensus terminology developed through the HL7 Gravity Project [15], and professional position statements [16].

Nurses conduct admission and ongoing assessments that may include living situation, family support, and social circumstances, and standardized nursing terminologies provide structures within which such information can be recorded [17]. Studies of clinician and staff experiences of social risk screening have reported time constraints, uncertainty about responsibility, and concern about what can be done with the information once collected [12,18]; screening tools also vary in their psychometric and pragmatic properties [19], and nursing leadership has been identified as a mechanism for embedding social needs work at the bedside [20]. We identified few studies focused specifically on nurses’ perspectives on SDOH documentation, and the available evidence examines mixed clinician groups, predominantly in North American settings [12,18].

In Saudi Arabia, health system reform has included substantial investment in health information technology, with high digital health maturity reported in governmental and private facilities [21] and high levels of EHR knowledge and use among healthcare providers [22]. Research on Saudi nurses’ EHR use has concentrated on usability, attitudes, and workflow integration [23,24] and on documentation time, with nurses in three hospitals spending a mean of 5.43 h per shift on electronic documentation [25], rather than on the content of what is recorded. The Saudi context also differs from the settings of most SDOH documentation research: expatriate nurses constitute a substantial proportion of the workforce [26], and cultural norms may influence how patients and professionals approach disclosure and documentation of sensitive social and financial information. What Saudi nurses record about patients’ social circumstances, and why, has not been examined. Whether the barriers reported in North American and European studies also arise where EHRs have been implemented recently, where social risk screening is not subject to an equivalent national reporting requirement, and where most responses to social need sit outside the health system has not been established.

This study explored registered nurses’ perspectives, experiences, and perceived barriers regarding the documentation of SDOH in EHRs in Saudi Arabia. Specifically, the study aimed to: (1) examine nurses’ understanding of SDOH and its perceived importance for patient care; (2) explore their experiences of documenting SDOH within EHR systems; (3) identify barriers and facilitators affecting SDOH documentation in clinical practice; and (4) describe their training needs and recommendations for improving documentation practices.

## 2. Materials and Methods

### 2.1. Design

A qualitative descriptive design was used [27]. Qualitative description stays close to participants’ accounts and produces a direct rendering of events and perspectives, which suited research questions concerned with how nurses experience and perceive documentation practice rather than with theory development [28]. Reporting follows the Consolidated Criteria for Reporting Qualitative Research (COREQ) 32-item checklist [29]; the completed checklist is provided in File S1.

Four related activities are distinguished throughout this paper. Identifying refers to a nurse becoming aware of a patient’s social circumstance through conversation, observation, or information from family, without necessarily using a structured instrument. Screening refers to the systematic application of a standardized question set or instrument to all patients, or to a defined patient group, generating a recorded result whether or not a need is present. Documenting refers to entering social information into the EHR, either in a structured field or as free text, and is the activity this study examined. Referring refers to initiating a formal request for another service or professional to respond to an identified need, which is recorded separately from the social information itself. The abbreviation SDOH is used for the conditions themselves, and the term social needs for specific unmet needs arising from those conditions in an individual patient. Because the study examined nurses’ perspectives and self-reported practice, statements about what is documented describe what participants reported doing rather than the measured content of the record; no audit or extraction of EHR data was undertaken.

### 2.2. Setting

The study was conducted with participants across five healthcare facilities in Saudi Arabia, comprising both governmental hospitals and private institutions and spanning primary, secondary, and tertiary levels of care. The facilities were located in the Riyadh and Al-Qassim regions, and all used EHR systems for clinical documentation. Local system configurations and institutional workflows varied across facilities. These contextual differences were considered when interpreting nurses’ accounts of SDOH documentation and the accessibility of relevant documentation fields within the EHR systems.

Participants were distributed across the five facilities as follows: Facility 1, *n* = 5; Facility 2, *n* = 4; Facility 3, *n* = 4; Facility 4, *n* = 3; Facility 5, *n* = 3. Facilities are identified by number rather than by name because the small number of nurses in some departments would otherwise make individual participants identifiable. Three facilities were governmental, and two were private. Of the five, three used a commercial integrated EHR platform, and two used a locally configured system. All five included structured nursing admission assessment forms; two provided a dedicated section or set of fields for social information, and in the remaining facilities, social information was recorded within general nursing notes.

### 2.3. Participants and Sampling

Purposive maximum-variation sampling was used to recruit registered nurses who could provide relevant perspectives on SDOH documentation across different clinical and professional contexts. Variation was sought in clinical specialty, years of clinical experience, healthcare sector, and nationality. Inclusion criteria were current employment as a registered nurse in a Saudi Arabian healthcare facility, at least one year of clinical experience, prior experience using EHR systems, and the ability to communicate in either Arabic or English. The final sample comprised 19 registered nurses. Three additional nurses were approached but declined to participate because of scheduling conflicts. Participant characteristics are presented in Table 1.

Recruitment was conducted from 6 August to 25 November 2025 through email invitations and professional networks. The first author and a research assistant from the department distributed the study invitations to eligible nurses. The researchers had no prior professional or supervisory relationship with the participants. All participants received information about the study and were informed that participation was voluntary and that they could decline or withdraw without consequences. The recruitment script is provided in File S3.

Recruitment and interviewing continued until the research team judged that additional interviews were contributing limited new material relevant to the study objectives and that the dataset provided sufficient information power in relation to the study aim, sample specificity, and analytic approach [30]. The research team therefore did not define or claim that theoretical or data saturation had been achieved [31]. This approach was consistent with the interpretive orientation of reflexive thematic analysis [32].

### 2.4. Data Collection

Data were collected through semi-structured interviews conducted between August and November 2025. The interviews were conducted by the first author (B.M.A.) and the second author (W.M.A.). An interview guide was developed based on the study objectives, relevant literature, and expert consultation and is provided in File S2. The guide covered participants’ professional background and experience with EHR systems, their understanding of SDOH, current documentation practices, perceived barriers and challenges, facilitators and supportive factors, training and education needs, the perceived use and impact of documented SDOH information, and recommendations for improving documentation practices. Probing questions were used to encourage elaboration and clarify participants’ meanings and experiences.

The interview guide was pilot-tested with two nurses who met the inclusion criteria. Data from the pilot interviews were not included in the final analysis, and minor wording changes were made to improve clarity and flow. Interviews were conducted in Arabic or English according to participant preference. Each interview lasted between 35 and 75 min, with a mean duration of 52 min. Of the 19 interviews included in the study, 10 were conducted face-to-face in private rooms at participants’ workplaces, and 9 were conducted through secure video conferencing, according to participant preference. No one other than the interviewer and participant was present during the interviews. All interviews were audio-recorded with participants’ consent.

Because two researchers conducted the interviews, several strategies were used to promote consistency between interviewers and to limit inter-interviewer variability. Both interviewers used the same interview guide, with a common opening statement, question sequence, and set of core probes, and both took part in the pilot interviews, which were used to agree on how each question would be introduced and followed up. After the first interviews, the two interviewers reviewed each other’s audio recordings and transcripts and discussed differences in probing depth, pacing, and responses to digressions. Interview conduct was then reviewed at regular research-team meetings throughout data collection, and topics that one interviewer had explored in depth were noted so that they could be followed up consistently in subsequent interviews. Both interviewers are fluent in Arabic and English, so language preference did not determine which interviewer conducted an interview. Consistent with the interpretive orientation of the study, the aim was comparable coverage of the guide across interviews rather than identical interview conduct [32].

The first and second authors transcribed the audio recordings. Field notes documenting contextual observations and reflective impressions were maintained throughout the data-collection period. No repeat interviews were conducted.

### 2.5. Researcher Characteristics and Reflexivity

The researchers directly involved in data collection and analysis were nurse academics with experience in nursing practice, nursing informatics, and qualitative research. The first and second authors, who conducted the interviews, are male registered nurses with doctoral qualifications and academic experience in nursing. Their clinical and academic experience informed their interpretation of nurses’ documentation practices and provided familiarity with the healthcare context in which the study was conducted.

Reflexivity was addressed throughout the research process. The researchers maintained individual reflexive journals to document their assumptions, methodological decisions, and developing interpretations. Regular research-team meetings were also conducted to discuss, compare, and critically challenge emerging interpretations. This process was intended to make researchers’ perspectives explicit and to support critical engagement with how their professional backgrounds and assumptions could shape data interpretation. Participants were informed of the researchers’ professional backgrounds and the purpose of the study before providing consent.

The study did not use a formal bracketing procedure to suspend or eliminate researchers’ preconceptions. Consistent with the reflexive thematic analysis approach, researcher subjectivity was acknowledged as an integral part of the interpretive process rather than treated as a source of bias that could be completely removed [32].

### 2.6. Data Analysis

Interviews were transcribed verbatim. Interviews conducted in Arabic were transcribed in the original language and subsequently translated into English by bilingual members of the research team. To assess translation accuracy, approximately 10% of the translated material was selected purposively to represent the range of interview content and independently back-translated by a bilingual member of the research team who was not involved in the initial translation. The back-translated segments were compared with the original Arabic transcripts, and discrepancies in meaning were reviewed and resolved through discussion. Transcripts were also checked against the original audio recordings before analysis.

Data were analyzed using reflexive thematic analysis [32], which was selected for its theoretical flexibility and compatibility with a qualitative descriptive design. Analysis followed the six phases originally described by Braun and Clarke [33]. Familiarization involved repeated reading of the transcripts and listening to the audio recordings while noting initial impressions. Coding was conducted across the full dataset, with two team members coding independently before comparing their interpretations and developing codes collaboratively. These discussions were used to broaden and deepen interpretation rather than to establish inter-coder agreement. Codes were then collated into candidate themes, and visual mapping was used to explore relationships among codes and potential themes. Candidate themes were reviewed against the coded extracts and the dataset as a whole and were merged, divided, refined, or discarded where appropriate. Themes were subsequently defined and named, and the analysis was written up using supporting participant extracts. Codes were developed inductively and organized into themes through collaborative discussion; no formal coding tree or predefined codebook was produced. Participant extracts are reported verbatim from the transcripts, with extracts from interviews conducted in Arabic presented in English translation. NVivo 14 (Lumivero, Denver, CO, USA) was used to support data management. An audit trail of analytic decisions was maintained throughout the analysis.

### 2.7. Trustworthiness

Trustworthiness was addressed using the criteria described by Lincoln and Guba [34]. Credibility was supported through independent coding, collaborative interpretation among team members, and member checking. Five participants were purposively selected from the study sample to provide variation in clinical specialty, years of nursing experience, and healthcare setting. This approach was used to obtain feedback from participants with different professional experiences relevant to SDOH documentation.

Member checking was conducted after the initial coding and development of the preliminary themes, but before the themes were finalized and Section 3 was completed. This timing allowed participant feedback to inform the refinement and interpretation of the emerging themes. The five participants were provided with the preliminary themes together with representative supporting extracts from the interview data and were asked to comment on whether the themes and interpretations accurately reflected their experiences. All five participants confirmed that the themes reflected their experiences, and their feedback was considered during the final refinement of the thematic structure and findings.

Transferability was supported through detailed description of the study context, participant characteristics, sampling and recruitment procedures, data-collection processes, and analytic procedures. Dependability was supported by maintaining an audit trail of methodological and analytic decisions throughout the study. Confirmability was supported through reflexive journaling, documentation of researcher positionality, collaborative discussion and critical examination of emerging interpretations, and grounding of interpretations in participant extracts.

### 2.8. Ethical Considerations

Ethical approval was granted by the Institutional Review Board of King Saud University, Riyadh, Saudi Arabia (KSU-HE-25-905) on 15 June 2025. All potential participants received written and verbal information about the purpose of the study, the procedures involved, the voluntary nature of participation, confidentiality arrangements, and their right to withdraw at any point without consequence. Informed consent was obtained from all participants before data collection. For face-to-face interviews, written informed consent was obtained before the interview, whereas electronic informed consent was obtained before remote interviews. The participant information sheet and informed consent form are provided in File S4.

Identifying information was removed from the transcripts, and participants were assigned alphanumeric codes (P01 to P19). Audio recordings and transcripts were stored on password-protected devices accessible only to the research team and will be retained for five years before secure deletion. All procedures were conducted in accordance with the Declaration of Helsinki.

## 3. Results

### 3.1. Participant Characteristics and Overview of Findings

Nineteen registered nurses participated in the study. Participant characteristics are presented in Table 1. The analysis generated four themes: (1) system design and interoperability; (2) time as a competing resource; (3) knowledge gaps and training needs; and (4) from documentation to action. The themes and corresponding subthemes are summarized in Table 2. Extracts from all 19 participants are presented across the four themes.

### 3.2. Theme 1: System Design and Interoperability

Participants described the EHR as an important part of the SDOH documentation process. Their experiences showed that the way social information was organized within the system could either make documentation easier or make it difficult to complete during routine care. Three related issues emerged: limitations in system design and interface, the balance between standardization and flexibility, and limited integration across care platforms.

#### 3.2.1. System Design and Interface Limitations

A common concern was that EHRs were mainly designed around clinical information, while social information was often harder to find or access. Participants described social history fields that were buried within menus or separated from the main nursing workflow.

P03 explained:

*“The social history is there, but honestly, you have to look for it. It’s kind of buried in the menus. When you’re really busy, it’s easy to just leave it because it’s not part of the main workflow.”*

P11 described another problem:

*“There are dropdowns for some of the social information, but the options are very limited. For housing, for example, you can say ‘owns’ or ‘rents,’ but what if the patient is homeless or staying with family? There’s really no option for that.”*

P07 said:

*“If there’s no specific field for it, I usually put it in the nursing notes. But then it’s just free text. You can’t really search for it or pull it into a report, so the information is kind of lost.”*

Overall, participants felt that the EHR could support SDOH documentation more effectively if relevant information were easier to find, more closely connected to routine nursing workflows, and available in formats that reflected patients’ actual circumstances.

#### 3.2.2. Standardization Versus Flexibility

Participants also discussed the challenge of finding the right balance between standardized documentation and the flexibility needed to describe individual patients. Standardized fields were viewed as useful because they could make information easier to record, compare, and analyze. At the same time, participants questioned whether fixed categories could adequately represent the complexity of patients’ social circumstances.

P15 explained:

*“I think we need standardized fields because otherwise it’s difficult to track the information or use it for research. But at the same time, every patient’s situation is different. A young single mother and an older person living alone could have completely different needs. It’s hard to put all of that into fixed categories.”*

P02 similarly described the checklist approach:

*“The hospital started using these SDOH questions last year. I think having them standardized is useful, especially for research, but sometimes it feels like you’re just going through a checklist. Some patients don’t seem very comfortable with that.”*

These accounts suggest that participants did not reject standardization. Rather, they wanted documentation systems that provided enough structure to support consistent recording while still allowing nurses to capture individual and sometimes complex social circumstances.

#### 3.2.3. Integration Across Care Platforms

Another concern was whether social information remained accessible as patients moved between departments or healthcare facilities. Participants described situations in which information documented during one encounter was not readily available during the next stage of care.

P09 said:

*“I document the social information when the patient is admitted, but sometimes when they move to another department or another hospital, that information isn’t there anymore. Different systems record different things, so some of it gets lost.”*

P11 added:

*“There isn’t really much continuity. What I document in the emergency department isn’t always visible in the outpatient clinic. Then we have to ask the patient the same questions again. It’s frustrating for them, and it takes more time.”*

Participants therefore viewed interoperability as more than a technical issue. For them, being able to carry social information across different points of care was important for avoiding repetition, maintaining continuity, and making previously documented information useful.

### 3.3. Theme 2: Time as a Competing Resource

Time emerged as one of the most practical challenges affecting SDOH documentation. Participants described having to balance social assessment and documentation with medications, clinical assessments, care planning, and other immediate nursing responsibilities. In busy settings, social information was often seen as important but less urgent than tasks directly related to the patient’s immediate clinical condition.

#### 3.3.1. Documentation Burden and Competing Priorities

Participants repeatedly returned to the amount of documentation already required in their daily work. When workloads were high, additional social assessment could feel difficult to fit in.

P04 said:

*“Honestly, it takes extra time to document social factors. If I have six patients and I’m already dealing with medications, assessments, and everything else, sometimes asking about housing or family support feels like something I just can’t get to.”*

P13 similarly explained:

*“There’s already a lot of documentation. We have vital signs, assessments, medications, physician orders, care plans; you have to document all of it. Then you add detailed social information on top of that. There’s only so much time.”*

These accounts show that the issue was not necessarily a lack of interest in SDOH. Rather, participants often had to decide where to spend limited time, particularly when several competing clinical demands were present.

#### 3.3.2. Workflow Integration Challenges

Participants also explained that SDOH assessment did not always fit naturally into established nursing workflows. This was particularly noticeable in acute care, where nurses described having to focus first on immediate clinical needs.

P11 explained:

*“In the emergency department, the priority is to stabilize the patient. Asking about their job or whether they have enough food at home doesn’t really fit into that moment. Maybe it should, but realistically, it’s difficult when everything is happening at once.”*

P06 described a similar problem during admission:

*“During admission, we’re supposed to collect that information, but sometimes we’re really busy, or the patient is too sick or tired to talk for long. So we document what we can and think, ‘I’ll come back to it later.’ But honestly, most of the time, that doesn’t happen.”*

Participants therefore described a gap between what the workflow expected them to document and what was realistically possible during a busy clinical encounter.

#### 3.3.3. Efficiency Versus Comprehensiveness

Participants recognized that working efficiently was necessary, but they also acknowledged that speed could come at the expense of understanding the patient’s full social situation.

P08 explained:

*“You learn to do quick assessments and cover the main things. With SDOH, you might ask, ‘Do you have family?’ or ‘Are you working?’ But you can miss a lot of the details. Someone can have family but no real support, or they can have a job and still not be able to afford their medications.”*

P14 reflected on the same issue:

*“I’ve become pretty efficient with documentation, but sometimes being efficient means you only get the basics. I know I’m not always capturing the whole picture of what’s happening in my patients’ lives.”*

Taken together, these accounts suggest that participants were aware of the trade-off between completing documentation efficiently and collecting enough detail to make the information clinically meaningful.

### 3.4. Theme 3: Knowledge Gaps and Training Needs

Participants’ experiences also pointed to uncertainty about what SDOH includes, what they were expected to document, and how detailed that documentation should be. Although most participants understood that social circumstances could affect health, they did not always have a clear or shared understanding of how this concept should translate into everyday nursing documentation.

#### 3.4.1. Understanding What Constitutes SDOH

Some participants were familiar with the term SDOH but were uncertain about its scope. They questioned whether their existing understanding included all of the factors that might influence patients’ health.

P10 said:

*“I’ve heard the term SDOH, but I’m still not completely sure what all it includes. Is it just housing and income? What about immigration status, or transportation, like whether the patient can actually get to their appointments?”*

P05 similarly explained:

*“When I think about social factors, I usually think about things like family support and where the patient lives. But when you say ‘social determinants of health,’ it sounds much broader. So I’m not always sure if what I’m documenting is actually what they mean by SDOH.”*

These accounts suggest that the challenge was not simply whether nurses recognized that social factors mattered. It was also whether they had a clear understanding of which factors should be assessed and documented.

#### 3.4.2. Uncertainty About Documentation Protocols

Participants also described uncertainty about when and where SDOH information should be entered into the EHR and how much detail was expected. In the absence of clear guidance, nurses often relied on their own judgment.

P12 explained:

*“There isn’t really a clear protocol that says, ‘These are the social factors you need to assess and document for every patient.’ So nurses do it differently. Some document a lot, and others hardly document anything.”*

P03 said:

*“Sometimes I’m not sure when I’m supposed to document it. Is it only during admission? What if I find something out later? Where am I supposed to put it? The guidelines aren’t really clear.”*

The absence of clear and consistent expectations appeared to contribute to variation in documentation practices across nurses.

#### 3.4.3. Training Deficits and Learning Needs

Participants described gaps in both their educational preparation and workplace training. One participant explained that nursing education had introduced the concept of social factors but had not provided enough practical guidance on assessment and documentation.

P01 said:

*“I think I need more training on how to actually document SDOH. We learned about social factors in nursing school, but it was mostly theory. We didn’t really learn how to assess them properly or document them in the EHR.”*

P17 described the situation during hospital orientation:

*“The orientation showed us how to use the EHR, like entering orders, documenting vital signs, and looking at patient records. But there wasn’t really anything about social information. It was kind of assumed that we would figure it out ourselves.”*

P16 explained what type of training would be most useful:

*“I’d really benefit from practical training. I’d like to see examples of what good SDOH documentation actually looks like, how to ask about things like income or housing without making the patient uncomfortable, and what to do with that information afterward.”*

Overall, participants were asking for practical rather than purely theoretical training, particularly examples of good documentation and guidance on how to discuss sensitive social issues with patients.

### 3.5. Theme 4: From Documentation to Action

Participants generally saw value in documenting SDOH, particularly when social information could help nurses understand patients’ needs and plan appropriate care. However, they also described frustration when documentation did not lead to any visible action. This created a tension between believing that SDOH information was important and questioning whether documenting it actually made a difference.

#### 3.5.1. Perceived Value for Patient Care

Participants gave clear examples of how social information could contribute to better and safer care. For some, understanding the social reasons behind a patient’s health behavior was essential to developing realistic care plans.

P07 explained:

*“I think documenting social determinants is really important. A patient might not be taking their medication because they don’t want to, but maybe they just can’t afford it. If we document that, we might be able to connect them with the right support.”*

P09 said:

*“Knowing what the patient’s situation is at home really helps with discharge planning. If someone lives alone and can’t manage their wound care, I need to know that. It’s not just extra information; it can actually affect whether the discharge is safe.”*

These accounts show that participants viewed SDOH documentation as clinically relevant when it helped explain patient needs and supported individualized care.

#### 3.5.2. Disconnect Between Documentation and Action

Despite recognizing its value, many participants questioned what happened after social information was entered into the record. For some, the uncertainty was whether anyone actually reviewed or acted on the information.

P18 said:

*“I document that the patient is having financial problems, but then what? Does anyone actually read it? Does anything change because I documented it?”*

P02 explained:

*“There’s a gap between documenting something and actually doing something about it. We might document that a patient doesn’t have enough food, but if there’s no social worker available or no clear referral process, the information just stays in the record.”*

P14 added:

*“Sometimes it feels like you’re documenting just because you have to. You tick the boxes, but then nothing really happens with the information. That can be pretty discouraging.”*

These accounts suggest that participants’ concerns were not only about whether SDOH information was documented, but also about whether the healthcare system had the resources and processes needed to act on that information.

#### 3.5.3. Motivation and Professional Responsibility

Participants also linked SDOH documentation to their understanding of nursing as holistic care. Some said they continued to document because they considered it part of their professional responsibility, even when they were uncertain about how the information would be used.

P05 explained:

*“I document it because I see it as part of holistic nursing care. We’re supposed to look at the whole patient, not just the disease. Even if nothing happens afterward, at least I’ve done my part.”*

P08 said:

*“What would make me document more is knowing that it actually leads to something. If I could see that my documentation helped a patient get transportation or housing support, I’d definitely pay more attention to those details.”*

P19 reflected on the broader meaning of nursing:

*“For me, it’s about whether we’re just treating the disease or actually caring for the person. I became a nurse because I wanted to help people. That means knowing something about their life, not just looking at their lab results. So, for me, documenting social factors is part of that.”*

These accounts suggest that nurses’ motivation to document SDOH was shaped not only by the EHR and workload but also by whether documentation aligned with their professional values and produced a visible benefit for patients.

### 3.6. Relationships Between Themes

The four themes were closely connected in participants’ accounts rather than operating as separate issues. Participants described how the design of the EHR could either facilitate or hinder SDOH documentation, while workload and competing clinical priorities affected the time available for social assessment. At the same time, uncertainty about what should be documented and how it should be recorded influenced nurses’ confidence in the process.

Participants also linked the value of documentation to what happened after information was entered into the record. When nurses believed that SDOH information could influence care, referrals, or patient outcomes, they described stronger motivation to document it. When the information appeared to remain unused, documentation could feel like another task added to an already demanding workload.

**Figure 1:**
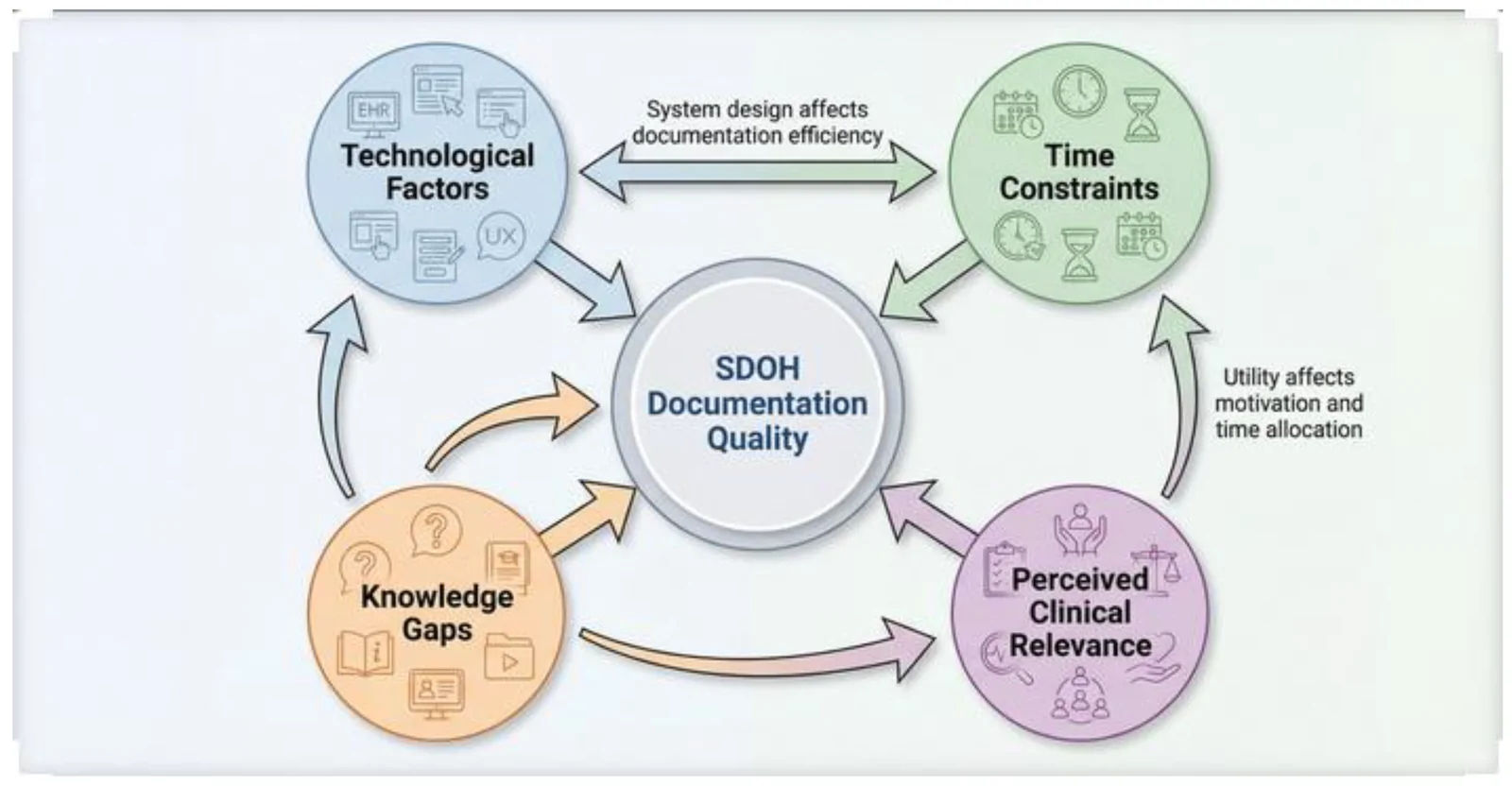
Interpretive model of the relationships between the four themes influencing SDOH documentation in EHRs. The model represents the research team’s reading of the interview data and was not tested empirically.

Figure 1 presents the research team’s interpretation of these relationships. The model illustrates how system-level factors, workflow pressures, knowledge and training, and perceived clinical value may interact to shape nurses’ SDOH documentation practices. The model represents an interpretation of the interview data and was not empirically tested.

## 4. Discussion

This study examined how registered nurses in Saudi Arabia experience the documentation of social determinants of health in electronic health records. Participants described technological, temporal, educational, and organizational conditions that together shaped what was recorded and how.

### 4.1. Technological Conditions of SDOH Documentation

The interface and structural limitations participants described are consistent with earlier work reporting that social information is inconsistently structured within EHRs and that the available fields often do not fit patients’ circumstances [9,10]. The tension participants described between standardized data elements and narrative flexibility corresponds to the design problem described in the informatics literature, in which social data must serve both analytic and individual-care purposes [10]. Evidence from Saudi settings points in the same direction: perceived usability predicts whether nurses integrate EHR use into their workflow [23].

The failure of social data to move between departments and facilities reflects long-standing interoperability challenges in health information exchange [35]. Technical standards such as HL7 FHIR are advancing data exchange capability [36], and the Gravity Project has developed consensus terminology specifically for social risk data [15], but implementation remains uneven, and awareness of such standards among the frontline nurses in this study appeared limited.

These findings suggest that system designers and implementers should treat SDOH functionality as a design requirement rather than an addition: placing social history fields within the clinical workflow rather than several screens away, offering structured elements that permit narrative qualification, and supporting transfer of social data across care settings. Involving nurses as end-users in system design may reduce the mismatch between system affordances and clinical reality [37].

### 4.2. Time and Documentation Workload

Time constraints were the barrier participants raised most consistently. The magnitude of the competing demand is documented in the Saudi context: nurses in three Saudi hospitals spent a mean of 5.43 h per shift on electronic documentation, significantly more than the 4.34 h reported by physicians in the same institutions [25]. Evidence from time-and-motion work indicates that electronic charting does not by itself increase documentation time relative to paper; nurses in one large US study spent about 19% of their time documenting regardless of whether electronic records were used [38]. Taken together, these findings suggest that the burden participants described derives less from the medium than from the accumulated volume of what is required, which is consistent with their account of social documentation as an addition to an already full workload rather than as part of it. Their prioritization of clinical over social documentation matches reports that competing demands lead staff to deprioritize social assessment [12].

The efficiency–comprehensiveness trade-off participants described has a practical consequence. Documentation that satisfies an audit requirement without capturing clinically usable detail produces information that, in P07’s account, cannot be searched or retrieved and is therefore effectively lost within the record. This suggests that adding SDOH fields to an EHR is unlikely on its own to improve data quality; workflow redesign is also required.

Approaches that might reduce the additional burden include embedding social questions at existing workflow touchpoints rather than creating separate assessment activities, collecting patient-reported information before the encounter through questionnaires or patient portals, although portal uptake is itself uneven among the populations most affected by social risk [39], and distributing responsibility across the team so that social workers or community health workers conduct detailed assessment where such roles exist. Screening without the organizational capacity to respond has been cautioned against on precisely these grounds [13]. Each of these requires organizational commitment and staffing that may not be available in every setting, and participants in this study described precisely that absence.

### 4.3. Knowledge, Training, and Professional Development

Participants’ uncertainty about what counts as an SDOH, how to assess it systematically, and where to record it points to a gap between how social determinants are taught and how they are practiced. Nursing education has historically addressed social determinants at the level of concept rather than clinical procedure, and calls have been made to strengthen the preparation of nurses to address health equity in practice [40].

Curricular change alone would not reach the practicing workforce. Participants asked for training with specific content: what factors are included and why, how to ask about income or housing without causing offense, where and when to record information in the particular system they use, and how documented information should inform care planning. This aligns with Saudi evidence that educational level and prior system exposure shape how nurses use EHRs [23,24]. The variability participants described, in which each nurse documents according to individual judgment, points to institutional protocols as a prerequisite for consistent data.

### 4.4. The Documentation-to-Action Gap

The most consequential finding of this study is the gap participants perceived between recording social information and anything following from it. Participants recognized the relevance of social factors while questioning whether their documentation influenced care or connected patients to services.

This concern is supported by quantitative evidence. In community health centers that implemented dedicated EHR tools for social data, nearly all screened patients had at least one social need recorded, yet only 19% had a corresponding referral documented [11]. Documented unmet social needs have also been associated with missed primary care appointments, indicating that identification alone does not resolve them [41]. Where documentation does not lead to action, it may come to be seen as an administrative exercise, reducing the motivation to document thoroughly, which in turn limits the value of the resulting data. Participants in this study described that sequence directly.

Interrupting it requires organizations to establish routes from identification to intervention: access to social work services for patients with identified needs, community resource information accessible at the point of care [16], decision support linked to specific social risk documentation, and feedback showing nurses what followed from what they recorded. Participants’ own accounts suggest that visible consequences would change their documentation behavior.

### 4.5. Convergence with International Evidence and Context-Specific Features

The barriers participants described, that is, interface limitations, competing workload, uncertain preparation, and an absent link between recording and response, closely resemble those reported in North American and European studies [7,11,12,13,18]. This convergence is informative rather than merely confirmatory. Most of that evidence comes from systems in which social risk screening is driven by payment and accreditation requirements and is delivered through long-established EHRs extended to accommodate social data; hospitals reporting to the United States Inpatient Quality Reporting Program, for example, have been required since 2024 to report screening across five health-related social need domains [42]. The facilities in this study operate under different conditions: EHRs were implemented recently and rapidly within national health system reform [21,43], and we are not aware of an equivalent national reporting requirement for social risk screening in place at the time of data collection. That the same barriers appear under these conditions suggests they are not products of legacy system architecture or of screening mandates, but of more general properties of how social information fits into clinical documentation.

Three features of the Saudi setting nevertheless change what these shared barriers mean locally. The first concerns where the route from documentation to action leads. In the systems that produced most of this evidence, responses to identified social needs are organized largely within healthcare, through hospital social work departments, care management, and referral to safety-net programs. In Saudi Arabia, responses are distributed across the state, the family, charitable organizations, and the market, and analysis of this care economy describes fragmented coordination between these sectors, no integrated referral pathway, and families carrying much of the unpaid caregiving [44]. Hospital social work exists but has been described as constrained by staffing, training, and role definition [45]. The accounts of P02 and P18, in which documented needs produced no visible response, are therefore consistent not only with the intra-organizational referral gap reported elsewhere but with an inter-sectoral one: information is recorded in a health record whose institutional counterpart lies outside the health system. Closing it would require interfaces between health facilities and social care providers, not only internal referral processes.

The second concerns who conducts the assessment and in which language. Non-Saudi nurses made up 42.1% of this sample, consistent with the composition of the national workforce [26], and EHR systems in these facilities are typically configured in English while many patient encounters take place in Arabic. Saudi evidence indicates that communication in linguistically diverse settings rests on individual language competence and informal peer support rather than on institutional language services [46], and non-Saudi staff have been reported to spend longer on electronic documentation than Saudi colleagues [25]. Social assessment is more language-dependent and more culturally situated than most clinical documentation, since questions about income, housing, and family support carry meanings that shift with the relationship between nurse and patient. P16’s request for guidance on asking about income without causing discomfort, and P02’s observation that patients appeared uneasy with a checklist format, point to this difficulty, although participants did not attribute it explicitly to language or nationality and the study was not designed to compare Saudi and non-Saudi accounts.

The third concerns what counts as a social risk and at what unit it should be recorded. Where extended family provides material and practical support, two patients with identical housing status may face very different circumstances depending on household composition, which is close to what P15 described in questioning whether fixed categories can represent individual situations. Financial difficulty is also disclosed differently, as it is treated as a family rather than an individual matter. Structured fields that record individual-level status may therefore capture the wrong unit of analysis in this setting, where family support capacity and dependants’ circumstances may carry more information. Fragmentation between governmental and private facilities, which national exchange platforms are intended to resolve but which remains incomplete [43], compounds the problem by leaving even well-recorded data unavailable at the next point of care, as P09 and P11 described.

These observations are interpretive. Participants were not asked about national policy, welfare arrangements, or nationality; no comparative data were collected, and the study was not designed to isolate context-specific determinants. Their function is to identify where this setting is likely to alter what a barrier means and what would resolve it, and to specify questions that comparative or multi-site work could test.

### 4.6. Mechanisms and Relative Weight of the Barriers

Identifying four barriers does not explain how they generate the documentation practices participants described. Three mechanisms are visible in the accounts and are summarized in the relationships shown in Figure 1.

The first is a filtering mechanism operating through system structure. Where a structured field existed, information entered it; where none existed, information moved into free-text nursing notes, which P07 described as unsearchable and impossible to pull into a report. Social information is therefore not simply omitted: part of it is recorded in a form that no downstream user, audit, or analysis can retrieve. This has a secondary effect, because information that cannot be retrieved cannot prompt a response, which participants then experience as evidence that documenting makes no difference. Interface design and the documentation-to-action gap are connected through this route rather than merely co-occurring.

The second is rationing by expected yield. Participants did not describe social documentation as unusually time-consuming in itself; they described choosing between competing tasks under time pressure (P04, P06). What settles that choice is the expected return on the time spent, and the accounts of P14 and P18 indicate a low expected return for social documentation relative to clinical tasks whose consequences are immediate and visible. Time scarcity is better understood as the medium through which a priority ordering is expressed than as an independent barrier, which is consistent with P08’s account that seeing results would change what they recorded. It also implies that releasing time alone would not redirect effort toward social documentation unless the expected yield changed with it.

The third is variability generated by the absence of a protocol. With no institutional specification of what to document, when, and in what detail (P03, P12), each nurse applied individual judgment, producing records that differ in scope and granularity for comparable patients. Heterogeneous data cannot support the aggregate uses, such as case-finding, reporting, or service planning, that would make documentation visibly consequential, which returns to the same gap. Training needs and the action gap are therefore linked through data quality rather than being separate problems.

These mechanisms imply an ordering that a list of barriers does not convey. In participants’ accounts, the documentation-to-action gap carries the widest effect, because it sets the value on which the other mechanisms operate: it determines the yield against which time is rationed, the purpose that protocols and training would serve, and whether recorded data are used at all. System design is the enabling condition, since response and feedback are possible only for information that can be retrieved. Protocols and training are the most immediately actionable, requiring institutional decision rather than system procurement or inter-sectoral coordination, but they affect the consistency of documentation rather than its consequence. A defensible sequence would therefore establish a route from record to response, structure the fields on which that route depends, and then prepare nurses to use them. This ordering is an interpretation of patterned accounts rather than a measured ranking: reflexive thematic analysis does not treat the frequency with which a topic is raised as a measure of its importance [32], and participants were not asked to rank the barriers they described. A design that asked them to do so, or that observed documentation behavior directly, could test it.

### 4.7. Interpretive Frameworks

Two frameworks help organize the findings, though neither guided the study design and both are applied retrospectively. From a sociotechnical systems perspective [47], SDOH documentation emerges from interaction between people, technology, organizational processes, and the wider environment, which suggests that interventions directed at any single element are unlikely to succeed alone. The Technology Acceptance Model [48] draws attention to perceived usefulness and perceived ease of use as determinants of system use; participants in this study described deficits in both, questioning the utility of what they recorded while describing systems that made recording difficult. Neither construct was measured here, so these frameworks are offered as interpretive lenses rather than as tested models.

### 4.8. Implications for Practice, Education, Policy, and Research

For healthcare organizations, the findings point to explicit documentation protocols specifying what, when, where, and how social information should be recorded; system-specific training; defined referral pathways connecting identified needs to services; feedback mechanisms showing nurses the downstream use of their documentation; and assessment of workload implications before new documentation requirements are introduced.

For system designers, the findings point to placing social history fields within the clinical workflow, combining structured elements with narrative capacity, supporting transfer of social data across care settings, and linking documentation to actionable prompts.

For nursing education, the findings point to teaching social assessment and documentation as clinical skills rather than concepts, including how to ask sensitive questions, how to record information in an EHR, how social information should inform care planning, and how to assess social circumstances across cultural differences. Continuing education is needed for nurses already in practice.

For policy, the findings point to support for standardized social data elements, funding for research on documentation and intervention strategies, and attention to the availability of the services to which identified needs would be referred.

Several of these implications take a specific form in Saudi Arabia. National platforms for health information exchange provide an existing route through which a minimum social data set, with Arabic value sets and definitions reflecting household and family support structures, could be standardized across governmental and private facilities rather than configured facility by facility [43]. Referral pathways would need to extend outside the health system to the social care and charitable providers that respond to most social need, where coordination is currently limited [44]. Where hospital social work capacity is constrained [45], specifying which needs generate a referral, to whom, and with what expected response matters more than adding screening items. Orientation for the large expatriate component of the workforce could address how social support is organized locally and how to raise these topics in Arabic, content that current arrangements leave to individual nurses to acquire informally [46].

For research, priorities include studies quantifying the prevalence and quality of SDOH documentation in Saudi healthcare settings, intervention studies testing strategies to improve documentation, studies of patients’ perspectives on disclosing social information to nurses, longitudinal work examining whether documented social information affects outcomes, comparative work across health systems, and implementation research on SDOH documentation initiatives. Mixed methods designs combining audit of documentation with qualitative exploration of context would be well suited to this area. Because this study captured a single professional perspective, designs that also include physicians, social workers, health information staff, managers, and patients would establish whether the barriers described here are perceived in the same way by those who would act on documented needs, and patient-facing work could examine what patients are willing to disclose, to whom, and under what circumstances.

### 4.9. Strengths and Limitations

The study addresses an underexplored topic in Saudi Arabia and other non-Western health systems. Variation in sector, specialty, experience, and nationality strengthened the range of perspectives, with extracts from all 19 participants represented. Analysis followed a defined procedure supported by an audit trail, member checking, and COREQ-guided reporting.

Several limitations should be considered. The study was conducted in one country, and findings should be interpreted within the specific EHR, organizational, and cultural context. Self-selection may have influenced the findings, as three invited nurses declined participation. Although researchers had no prior professional or supervisory relationships with participants, the nurse-academic research team may have influenced participants’ responses. Data were self-reported rather than directly observed, and social desirability or recall may have affected accounts. Interviews were conducted at a single time point and therefore do not capture changes over time. Interviews were conducted in Arabic or English, and Arabic transcripts were translated into English for analysis, with approximately 10% of the translated material independently back-translated; some linguistic nuances may therefore have been lost. Finally, interpretation was shaped by the research team’s professional and contextual knowledge, with reflexive journaling and team discussions used to support critical reflection. The study also captured a single professional perspective. Nurses record social information, but acting on it involves physicians, social workers, case managers, and administrators, and patients are its source. The documentation-to-action gap in particular is described here from one side: participants could report that they saw no response to what they recorded, but not whether a response occurred outside their view or why it did not. The findings therefore represent nurses’ accounts of a multi-actor process rather than an account of that process as a whole, and the contextual and mechanistic interpretations offered in Section 4.5 and Section 4.6 require corroboration from other stakeholder groups and from documentation audit.

## 5. Conclusions

Registered nurses in this study regarded social determinants of health as relevant to patient care but described conditions that made systematic documentation difficult. Social history fields were hard to locate and rigidly structured, social data did not follow patients across settings, documentation competed with a clinical workload that took precedence, preparation for social assessment was limited, and recorded information seldom appeared to lead to any action.

These findings suggest that improving SDOH documentation requires changes at more than one level: EHR design that places social data within the clinical workflow, institutional protocols that define expectations, training that addresses assessment and documentation as clinical skills, and referral pathways that connect identified needs to available services. The last of these may be the most consequential, since participants’ willingness to document depended on whether they believed anything followed from it. As a qualitative study conducted in one country with 19 participants, these findings describe the perspectives of this group rather than the prevalence or quality of documentation practice in Saudi Arabia, which would require separate investigation. The contribution of this study lies less in the barriers themselves, which largely match those reported in North America and Europe, than in their persistence in a system with recently implemented EHRs and no equivalent screening mandate, in the location of most responses to social need outside healthcare, and in the account offered here of how the barriers reinforce one another.

## Figures and Tables

**Table 1:** Participant characteristics (*N* = 19).

Characteristic	*n* (%)
Gender	
Female	15 (78.9)
Male	4 (21.1)
Age group	
<25 years	2 (10.5)
25–34 years	9 (47.4)
35–44 years	6 (31.6)
≥45 years	2 (10.5)
Years of nursing experience	
<3 years	3 (15.8)
3–5 years	5 (26.3)
6–10 years	6 (31.6)
>10 years	5 (26.3)
Healthcare sector	
Governmental	12 (63.2)
Private	7 (36.8)
Clinical department	
Medical-surgical	6 (31.6)
Emergency	3 (15.8)
Intensive care	3 (15.8)
Outpatient/primary care	4 (21.1)
Pediatrics	2 (10.5)
Obstetrics/gynecology	1 (5.3)
Nationality	
Saudi	11 (57.9)
Non-Saudi	8 (42.1)

Note: Percentages are calculated using *N* = 19 and are rounded to one decimal place; therefore, totals may differ slightly from 100.0% because of rounding.

**Table 2:** Themes and subthemes.

Theme	Subthemes
1. System design and interoperability: technological facilitators and barriers	1.1 System design and interface limitations;
	1.2 Standardization versus flexibility;
	1.3 Integration across care platforms.
2. Time as a competing resource: documentation demands and workflow	2.1 Documentation burden and competing priorities;
	2.2 Workflow integration challenges;
	2.3 Efficiency versus comprehensiveness.
3. Knowledge gaps and training needs: understanding what to document	3.1 Understanding what constitutes SDOH;
	3.2 Uncertainty about documentation protocols;
	3.3 Training deficits and learning needs.
4. From documentation to action: clinical relevance and utilization	4.1 Perceived value for patient care;
	4.2 Disconnect between documentation and
	action;
	4.3 Motivation and professional responsibility.

## Data Availability

The datasets collected and analyzed during the current study are not publicly available because of confidentiality protections for participants but are available from the corresponding authors on reasonable request, subject to ethical approval and a data sharing agreement.

## Supplementary Materials

The following supporting information can be downloaded at: https://www.mdpi.com/article/10.3390/healthcare14213676/s1. File S1: Consolidated Criteria for Reporting Qualitative Research (COREQ): 32-Item Checklist; File S2: Semi-structured interview guide; File S3: Participant recruitment script; File S4: Participant information sheet and informed consent form.
