# Supplementary material for: Registered Nurses’ Perspectives on Documenting Social Determinants of Health in Electronic Health Records: A Qualitative Descriptive Study in Saudi Arabia
Source: Healthcare (Basel). 2026 Oct 30;14(21):3676. doi: 10.3390/healthcare14213676 (PMC13763305; doi:10.3390/healthcare14213676)
Supplement: Supplementary file 1 [file healthcare-14-03676-s001.zip › healthcare-4572286-supplementary.pdf]

Supplementary Materials

This file contains the supplementary materials referenced in the manuscript:

- File S1: COREQ 32-item checklist
- File S2: Semi-structured interview guide
- File S3: Participant recruitment script
- File S4: Participant information sheet and informed consent form

File S1. Consolidated Criteria for Reporting Qualitative Research (COREQ): 32-Item Checklist

Reference: Tong, A.; Sainsbury, P.; Craig, J. Consolidated criteria for reporting qualitative research (COREQ): a 32-item checklist for interviews and focus groups. *Int. J. Qual. Health Care* **2007**, *19*, 349–357.

No.	COREQ item	Response	Where addressed
**Domain 1: Research team and reflexivity**			
1	Interviewer/facilitator	B.M.A. and W.M.A. (first and second authors); strategies to promote consistency between the two interviewers are described	Section 2.4
2	Credentials	Registered nurses with doctoral qualifications	Section 2.5
3	Occupation	Nurse academics	Section 2.5
4	Gender	Both interviewers (B.M.A., W.M.A.) are male	Section 2.5
5	Experience and training	Nursing practice, nursing informatics, and qualitative research	Section 2.5
6	Relationship established prior to study	No prior professional or supervisory relationship with participants	Section 2.3
7	Participant knowledge of the interviewer	Researchers' professional backgrounds and the study purpose were explained before consent	Section 2.5
8	Interviewer characteristics and reflexivity	Reflexive journals, research-team meetings, documented positionality; no bracketing procedure used	Section 2.5
**Domain 2: Study design**			
9	Methodological orientation and theory	Qualitative descriptive design; reflexive thematic analysis	Sections 2.1, 2.6
10	Sampling	Purposive maximum-variation sampling	Section 2.3

No.	COREQ item	Response	Where addressed
11	Method of approach	Email invitations and professional networks, distributed by the first author and a departmental research assistant; recruitment script in File S3	Section 2.3
12	Sample size	19 registered nurses	Sections 2.3, 3.1
13	Non-participation	Three nurses approached declined because of scheduling conflicts	Section 2.3
14	Setting of data collection	Ten interviews face-to-face in private rooms at participants' workplaces; nine by secure video conferencing	Section 2.4
15	Presence of non-participants	No one other than the interviewer and participant was present	Section 2.4
16	Description of sample	Demographic characteristics reported in Table 1	Section 3.1, Table 1
17	Interview guide	Semi-structured guide developed from objectives, literature, and expert consultation; pilot-tested with two nurses whose data were excluded	Section 2.4; File S2
18	Repeat interviews	No	Section 2.4
19	Audio/visual recording	All interviews audio-recorded with participants' consent	Section 2.4
20	Field notes	Field notes on contextual observations and reflective impressions maintained throughout data collection	Section 2.4
21	Duration	35–75 min; mean 52 min	Section 2.4
22	Data saturation	Not claimed. Recruitment was judged against information power, consistent with reflexive thematic analysis	Section 2.3
23	Transcripts returned	No. Full transcripts were not returned to participants; preliminary themes with supporting extracts were returned to five participants for member checking	Sections 2.4, 2.7
**Domain 3: Analysis and findings**			
24	Number of data coders	Two research-team members coded independently before collaborative development of codes	Section 2.6
25	Description of the coding tree	No. Codes were developed inductively and organised into themes through	Section 2.6

No.	COREQ item	Response	Where addressed
		collaborative discussion; no formal coding tree or predefined codebook was produced	
26	Derivation of themes	Themes were constructed inductively through the six phases of reflexive thematic analysis	Section 2.6
27	Software	NVivo 14 (Lumivero, Denver, CO, USA), used for data management	Section 2.6
28	Participant checking	Five participants, purposively selected for variation in specialty, experience, and setting, reviewed preliminary themes with supporting extracts after initial coding and before themes were finalised	Section 2.7
29	Quotations presented	Verbatim extracts from all 19 participants are presented, identified by participant code (P01–P19). Extracts from interviews conducted in Arabic are presented in English translation	Section 3
30	Data and findings consistent	Each theme and subtheme is supported by verbatim participant extracts drawn directly from the transcripts	Section 3
31	Clarity of major themes	Four major themes are reported, each with its own subsection and summarised in Table 2	Section 3, Table 2
32	Clarity of minor themes	Twelve subthemes are reported, three under each major theme	Section 3, Table 2

File S2: Semi-Structured Interview Guide

Title: Registered Nurses' Perspectives on Documenting Social Determinants of Health in Electronic Health Records: A Qualitative Descriptive Study in Saudi Arabia

Introduction: Thank you for agreeing to participate in this research study. This interview will explore your experiences and perspectives regarding the documentation of social determinants of health in electronic health records. There are no right or wrong answers, we are interested in your personal views and experiences. The interview will last approximately 45–60 minutes, although the actual duration may be shorter or longer depending on how much you wish to share. Please remember that your participation is voluntary, and you may choose not to answer any question or stop the interview at any time.

Do you have any questions before we begin?

Section 1: Background and Experience

1. Could you tell me about your role as a nurse and your experience with electronic health record systems?
   - *Probe:* How long have you been using EHR systems?
   - *Probe:* Which EHR system do you currently use?
2. In your day-to-day work, what types of information do you typically document in the electronic health record?

Section 2: Understanding of Social Determinants of Health

3. When you hear the term "social determinants of health," what comes to mind?
   - *Probe:* What do you understand this term to mean?
   - *Probe:* Can you give examples of factors you would consider social determinants?
4. In your view, how important are social factors in influencing patients' health outcomes?
   - *Probe:* Can you think of situations where social factors affected patient care?

Section 3: Current Documentation Practices

5. Could you describe how you currently document social information about patients in the EHR?
   - *Probe:* What specific information do you document?
   - *Probe:* Where in the EHR do you record this information?
   - *Probe:* How often do you document social factors?
6. Can you walk me through a recent example where you documented social information about a patient?
   - *Probe:* What prompted you to document this information?
   - *Probe:* How did you gather this information from the patient?

Section 4: Barriers and Challenges

7. What challenges or barriers do you face when trying to document social determinants of health in the EHR?
    - *Probe:* Are there technological barriers related to the EHR system?
    - *Probe:* Are there time constraints?
    - *Probe:* Are there knowledge or training gaps?
8. Are there specific social factors that you find particularly difficult to document? Why?
9. How does documenting social information fit into your overall workflow and competing demands?

Section 5: Facilitators and Supportive Factors

10. What factors help or support you in documenting social determinants of health?
    - *Probe:* Are there features of the EHR that facilitate this?
    - *Probe:* Are there organizational supports or policies?
11. Are there colleagues or other healthcare professionals who influence how you document social information?

Section 6: Training and Education

12. What training have you received related to assessing and documenting social determinants of health?
    - *Probe:* In your nursing education?
    - *Probe:* In workplace training or orientation?
13. What additional training or education would help you document social factors more effectively?

Section 7: Use and Impact of Documented Information

14. To what extent do you believe the social information you document is actually used in clinical decision-making?
    - *Probe:* By yourself?
    - *Probe:* By other healthcare providers?
15. Can you describe a situation where documented social information influenced patient care?
16. What motivates you to document social determinants of health?
    - *Probe:* What would increase your motivation?

Section 8: Recommendations and Future Directions

17. If you could change anything about how social determinants are documented in your workplace, what would you change?

18. What recommendations would you make to improve SDOH documentation in nursing practice?

19. Is there anything else about documenting social determinants of health that you would like to share?

Closing: Thank you very much for your time and valuable contributions to this research. Your perspectives will help us better understand how to improve SDOH documentation in nursing practice.

File S3: Participant Recruitment Script

Title: Registered Nurses' Perspectives on Documenting Social Determinants of Health in Electronic Health Records: A Qualitative Descriptive Study in Saudi Arabia

Mode of delivery: Email invitation and professional networks, distributed by the first author and a departmental research assistant.

Subject: Invitation to take part in a research study on documenting social determinants of health

Dear Colleague,

You are invited to take part in a research study conducted by researchers from the College of Nursing, King Saud University. The study explores registered nurses' perspectives on documenting social determinants of health (SDOH), such as housing, income, family support, and transportation, in electronic health records (EHRs).

Who can take part?

You are eligible to take part if you:

- are currently employed as a registered nurse in a healthcare facility in Saudi Arabia;
- have at least one year of clinical experience;
- have experience using an EHR system; and
- can communicate in Arabic or English.

What does taking part involve?

Taking part involves one individual interview lasting approximately 45–60 minutes. The interview can be conducted in Arabic or English, face-to-face in a private room at your workplace or through secure video conferencing, at a time convenient for you. With your permission, the interview will be audio-recorded.

Is taking part voluntary?

Yes. Your decision whether to take part will not affect your employment or professional standing, and you may withdraw at any time without giving a reason. The research team has no professional or supervisory relationship with the nurses being invited.

Will my information be kept confidential?

Yes. Your name and workplace will not appear in any report or publication. Interview transcripts will be de-identified and assigned a participant code.

Ethical approval

This study has been approved by the Institutional Review Board of King Saud University (KSU-HE-25-905).

How can I take part or find out more?

If you are interested in taking part or would like more information, please reply to this email or contact Dr. Bader M. Almutairy (abader@ksu.edu.sa). You will receive the participant information sheet and consent form (File S4) before an interview is arranged.

Thank you for considering this invitation.

On behalf of the research team, College of Nursing, King Saud University

File S4: Participant Information Sheet and Informed Consent Form

Study title: Registered Nurses' Perspectives on Documenting Social Determinants of Health in Electronic Health Records: A Qualitative Descriptive Study in Saudi Arabia

Research team: Dr. Bader M. Almutairy and Dr. Waleed M. Alshehri (interviewers), with Dr. Abdulaziz M. Alodhailah, Dr. Mohammed Almutairi, and Dr. Thurayya Eid, College of Nursing, King Saud University, Riyadh, Saudi Arabia

Ethical approval: Institutional Review Board, King Saud University (KSU-HE-25-905)

Part A. Participant information sheet

1. Purpose of the study. This study explores how registered nurses understand, experience, and document social determinants of health (SDOH) in electronic health records, including the barriers they face and the training and support they need.

2. What will happen if I take part? You will be asked to take part in one semi-structured interview lasting approximately 45–60 minutes, in Arabic or English according to your preference. The interview will take place face-to-face in a private room at your workplace or through secure video conferencing. With your permission, the interview will be audio-recorded and later transcribed. Some participants may also be invited to comment on a summary of the preliminary findings; this is optional.

3. Possible risks or discomforts. The study involves minimal risk. Some questions ask about documentation practices in your workplace, which you may find sensitive. You may decline to answer any question, pause the interview, or stop at any time.

4. Possible benefits. There is no direct benefit to you from taking part. Your views may help improve how social information is documented and used in nursing practice.

5. Confidentiality. Identifying information will be removed from transcripts, and you will be assigned a participant code. Your name and workplace will not appear in any report or publication. Short anonymized quotations from your interview may be included in publications. Audio recordings and transcripts will be stored on password-protected devices accessible only to the research team and will be retained for five years before secure deletion.

6. Voluntary participation and withdrawal. Taking part is voluntary. You may withdraw at any time without giving a reason and without any consequence for your employment or professional standing.

7. Contact information. For questions about the study, please contact Dr. Bader M. Almutairy (abader@ksu.edu.sa) or Dr. Thurayya Eid (teid@ksu.edu.sa). For questions about your rights as a research participant, you may contact the Institutional Review Board of King Saud University.

Part B. Informed consent form

Please confirm each statement:

- I have read (or had read to me) the participant information sheet and have had the opportunity to ask questions.
- I understand that my participation is voluntary and that I may withdraw at any time without giving a reason and without consequence.
- I agree to the interview being audio-recorded.

- I understand that anonymized quotations from my interview may be used in reports and publications.
- I agree to take part in this study.

Participant name: ______________________________

Participant signature: ___________________________ Date: ____________

Researcher name: _______________________________

Researcher signature: ___________________________ Date: ____________

Electronic consent (remote interviews): Participants interviewed through video conferencing received this information sheet in advance and confirmed each statement above electronically before the interview began.
